# The Plasticizer Dibutyl Phthalate (DBP) Impairs Pregnancy Vascular Health: Insights into Calcium Signaling and Nitric Oxide Involvement

**DOI:** 10.3390/jox15040127

**Published:** 2025-08-06

**Authors:** Ana R. Quelhas, Melissa Mariana, Elisa Cairrao

**Affiliations:** 1 RISE-Health, Department of Medical Sciences, Faculty of Health Sciences, University of Beira Interior, Av. Infante D. Henrique, 6200-506 Covilhã, Portugal; ana.rita.quelhas@ubi.pt; 2 FCS-UBI, Faculty of Health Sciences, University of Beira Interior, 6200-506 Covilhã, Portugal

**Keywords:** phthalate exposure, endocrine disrupting compound, vascular dysfunction, vascular smooth muscle cells, pregnancy exposome, fetoplacental disruption, nitric oxide pathway, calcium channels

## Abstract

Dibutyl phthalate (DBP) is used as a plasticizer to enhance flexibility in several household products, cosmetics, and food-contact materials. Due to its harmful effects, DBP is restricted or banned in children’s products and food items, particularly in Europe. Due to its endocrine disruptor properties and considering its ability to cross the placental barrier, it is imperative to study DBP’s vascular effects in pregnancy, given the vulnerability of this period. Thus, this study investigated the potential effects of DBP on the cardiovascular system using umbilical arteries from healthy pregnant women. Specifically, the impact of DBP on the vascular reactivity after both rapid and 24 h DBP exposure was analyzed, as well as the contractility and the cell viability of vascular smooth muscle cells (VSMC). DBP did not exhibit overt cytotoxic effects on VSMCs, possibly due to its adsorption onto polystyrene surfaces, potentially limiting bioavailability. Interestingly, DBP induced vasorelaxation in a concentration-dependent manner. Although mechanistic insights remain to be fully elucidated, the results suggest the involvement of pathways associated with nitric oxide signaling and calcium handling. Overall, DBP exposure appears to modulate arterial tone regulation, which may have implications for vascular function during pregnancy.

## 1. Introduction

An endocrine-disrupting chemical (EDC) is defined by the U.S. Environmental Protection Agency (EPA) as “an agent that interferes with the synthesis, secretion, transport, binding, or elimination of natural hormones in the body that are responsible for the maintenance of homeostasis, reproduction, development and/or behavior” [1,2]. EDCs exert their effects through diverse mechanisms, including mimicking or antagonizing endogenous hormones, altering hormone synthesis or metabolism, and interfering with hormone receptor signaling pathways [3,4]. While traditionally their actions have been associated with nuclear hormone receptors, growing evidence highlights their interaction with non-classical receptors, such as membrane-bound estrogen or androgen receptors, which mediate rapid and tissue-specific hormonal responses [3,5]. These complex mechanisms can contribute to a wide range of health consequences, extending beyond reproductive or developmental disorders to include metabolic syndromes, neurodevelopmental abnormalities, immune dysregulation, and certain cancers [4,6]. Therefore, a more detailed understanding of the multifaceted biological impacts of EDCs is essential to comprehensively assess their risks to human and environmental health.

Fetal development is particularly sensitive to the effects of endocrine disruptors, which can result in harmful consequences that may not be immediately recognized but can be manifested later in life. Importantly, exposure to these contaminants can also affect future generations, resulting in transgenerational health impacts [7,8,9].

Many chemicals are recognized as endocrine disruptors, including phthalates. These compounds are diesters derived from phthalic acid and are generally classified into two groups: high molecular weight (HMW) and low molecular weight (LMW) [10,11]. HMW phthalates are commonly used as plasticizers in products like polyvinyl chloride (PVC) as well as in various applications including plastics, food packaging, processing materials, and vinyl toys [11,12]. Among them, di (2-ethylhexyl) phthalate (DEHP) is the most extensively studied and widely used. In contrast, LMW phthalates are primarily found in personal care products. Notable examples include diethyl phthalate (DEP) and dibutyl phthalate (DBP) [11,12]. DBP is the most extensively investigated low molecular weight phthalate. Mostly used as a plasticizer, it has been found in home furnishings, car care products, toys, cosmetics, and food-contact materials. However, due to its endocrine disruption properties and adverse effects already demonstrated, DBP has been extensively restricted and banned from children care and food products, mainly in Europe [13,14].

In the human body, DBP is initially hydrolyzed into its primary metabolite, monobutyl phthalate (MBP), which is then excreted in urine [12]. This compound has been detected in various biological samples from the general population, including urine [15], serum [16], semen [17], and even nails [18]. Additionally, it has been found in maternal urine [19,20], maternal serum [21], breast milk [11], and amniotic fluid [22].

Recent animal studies have indicated that DBP may contribute to the development of several reproductive, metabolic, nervous, and cardiovascular disorders [23]. However, its effects on humans require further research for a better understanding. Nevertheless, epidemiological studies have suggested that DBP may lead to lung function and airway impairment [24,25], and it may influence the absorption and cellular distribution of thyroid hormone, thereby disrupting the thyroid axis [6,26,27], which in turn may lead to developmental and reproductive issues [28], has a positive association with subclinical atherosclerosis and coronary heart disease [29,30], and can contribute to a decrease in heart rate variability [31].

Thus, the present study aimed to investigate how exposure to DBP may impair vascular homeostasis in pregnant women. Considering that the umbilical cord is the vital maternal–fetal connection, to achieve this goal, human umbilical arteries (HUAs) were used. In addition to being easily obtained, these serve as an excellent model to study the vascular implications of EDCs in pregnant women. In addition, HUA smooth muscle cells (HUASMCs), derived from the umbilical artery, are key regulators of fetal–placental blood flow through the modulation of their contractile activity [32,33].

## 2. Materials and Methods

### 2.1. Ethics Statements

For this study, all biological samples were obtained from the obstetrics unit of “Unidade Local de Saúde da Cova da Beira” (ULS Cova da Beira; Covilhã, Portugal). All procedures performed with these samples were approved by the Ethics Committee of the ULS Cova da Beira (No.33/2018, 18 July 2018) and according to the Declaration of Helsinki principles. All pregnant women gave written informed consent.

### 2.2. Sample Collection

The forty-two human umbilical cords (HUCs) used in this study were collected from vaginal deliveries of full-term pregnancies. The selected pregnant women had no pathologies during pregnancy and those who used medications other than folic acid, iron supplements, or other vitamins during their pregnancy, were excluded from the study.

The umbilical cords were cut from the proximal half to the newborn (20 cm) and collected in tubes containing sterile physiological saline solution [PSS; composition: NaCl 110 mM; CaCl_2_ 0.15 mM; KCl 5 mM; MgCl_2_ 2 mM; HEPES 10 mM; NaHCO_3_ 10 mM; KH_2_PO_4_ 0.5 mM; NaH_2_PO_4_ 0.5 mM; Glucose 10 mM and EDTA 0.5 mM]. To prevent possible contamination, an antibiotic–antimycotic solution was added, consisting of penicillin (5 U/mL), streptomycin (5 μg/mL), and amphotericin B (12.5 ng/mL). The samples were stored at 4 °C until use, within a timeframe of 4 to 24 h. Specifically, 27 HUCs were used for the organ bath technique, while 15 HUCs were used for cell culture of HUASMCs. A table with selection criteria of the HUCs, namely the excluded pathologies, has been included in the Supplementary Material (Table S1).

### 2.3. Tissue Preparation

HUAs were used for arterial contractility studies, following the protocol described by Saldanha et al. [34].

All experiments were conducted on arteries without endothelium to investigate the effects of DBP on smooth muscle contractility, thereby eliminating any interference from the endothelium. Initially, the HUAs were isolated from the Wharton’s jelly, then, the endothelium was mechanically removed, and the arteries cut into 3 to 5 mm rings and placed in DMEM-F12 [Dulbecco’s Modified Eagle’s Medium/Nutrient Mixture F-12 Ham supplemented with NaHCO_3_ (1.2 g/L) and L-ascorbic acid (20 mg/L)].

### 2.4. Contractility Experiments in HUA Rings

The organ bath technique was used to analyze the direct/short-term (non-genomic) effects and the long-term (genomic) effects of DBP on HUAs. After 24 h, the rings were placed in the organ bath with a Krebs bicarbonate solution (Krebs; composition: KCl 5.0 mM; EDTA 0.03 mM; MgSO_4_·7H_2_O 1.2 mM; KH_2_PO_4_ 1.2 mM; L-ascorbic acid 0.6 mM; CaCl_2_ 0.5 mM; NaCl 119 mM; NaHCO_3_ 25 mM and glucose 11 mM) at a temperature of 37 °C and in continuous contact with carbogen gas (95% O_2_ and 5% CO_2_). To measure the tension, the HUA rings were suspended between two parallel stainless-steel wires and placed into the organ bath chamber (LE01.004, Letica, Madrid, Spain).

Initially, the arterial rings were placed under a baseline tension of 2.0 to 2.5 g and allowed to equilibrate for 45 min. The viability of the rings was then assessed by exposing them to serotonin (5-HT; 1 μM). For the studies examining direct vascular effects, only arterial rings that contracted more than 1 g were included. Then, to understand the direct vascular effects of DBP on HUA contractility, different concentrations of DBP (0.001; 0.01; 0.1; 1; 10; 100; 500; and 1000 μM) were added incrementally over baseline tension. These concentrations of DBP were selected based on prior studies conducted by the research group on other phthalates, specifically DEP and DEHP, and the non-monotonic dose–response curve typical of EDCs [35,36]. In addition, considering the DBP quantification in human blood that ranged from 0.0224 µg/L (0.000099 µM) in Danish pregnant women [37] and 150.83 µg/L (0.542 µM) in traffic policeman from China [38], the in vitro–in vivo scaling factor was also considered [39,40], and as such the selected concentrations in this study ranged from lower and beyond 200-fold the maximum concentration (0.001–1000 µM). Afterwards, the rings were subsequently contracted using 5-HT (1 μM) and potassium chloride (KCl; 60 mM), and upon stabilization of the contractile response, the same concentrations of DBP were introduced. Control experiments were conducted using vehicle-matched ethanol concentrations corresponding to those present in each DBP treatment, ensuring that the amount of ethanol was identical across all treatment and control conditions.

The genomic effects of DBP were analyzed by incubating the arteries for 24 h with 0.001, 0.1, and 100 μM of DBP. Arterial rings were then contracted using 5-HT (1 μM) or KCl (60 mM). After stabilization of the response to these contractile agents, incremental concentrations of nifedipine (Nif; 0.1, 1, and 10 μM), or sodium nitroprusside (SNP; 0.1, 1, 10, and 100 μM) were added. This procedure was performed in the absence of light, as both Nif and SNP are sensitive to photodegradation. Similarly, the control experiments were carried out using untreated (no incubation) and treated conditions, in which the percentage of ethanol (0.01%) corresponded to the maximum concentration of the DBP treatment. All incubations were carried out in glass test tubes. The DBP concentrations used to analyze the genomic effects were chosen to include a wider range, including a high concentration (100 μM), an intermediate concentration (0.1 μM), and a low concentration (0.001 μM).

### 2.5. Smooth Muscle Cells Isolation

Smooth muscle cells were isolated from human umbilical arteries according to the method described by Saldanha et al. [34]. After isolation of the arteries, small arterial rings were cut into rectangular pieces to expose the arterial lumen. The vascular endothelium was mechanically removed using a sterile cotton bud and layers of vascular smooth muscle from the tunica media were extracted and plated in collagen-coated culture dishes and maintained at 37 °C in an atmosphere of 95% air and 5% CO_2_. The culture medium used to promote cell growth consisted of DMEM-F12 supplemented with 5% fetal bovine serum (FBS), 0.25% bovine serum albumin (BSA), 5 μg/mL epidermal growth factor (EGF), 0.5 ng/mL fibroblast growth factor (FGF), 2 μg/mL heparin, and 5 μg/mL insulin. Additionally, a mixture of antibiotics was included, with 5 U/mL penicillin, 5 μg/mL streptomycin, and 12.5 ng/mL amphotericin B. The culture medium was renewed every 2 to 3 days. On average, confluent primary cultures were obtained within 30 days. Subcultures were performed for up to 4 to 5 passages, and the cells were used for cell viability studies and for assessing cell contractility.

### 2.6. MTT Assay

The MTT test (3-[4,5-dimethylthiazol-2-yl]-2,5-diphenyltetrazolium bromide) is employed to evaluate cell viability, proliferation, and cytotoxicity in vitro. The colorimetric assay measures the reduction in tetrazolium salt (MTT) into purple formazan crystals by cellular dehydrogenase enzymes from living cells. Thus, the ability to produce these crystals serves as an indicator of cell viability. This assay was conducted following the procedure outlined by Mariana et al. [41].

When the HUASMCs reached confluence, they were exposed to various concentrations of DBP (0.0001; 0.001; 0.01; 0.1; 1; 10; 100; 500; 1000 μM), solvent (ethanol—0.1% and 0.05%), and culture medium for 24 h. At the end of this incubation period, in the absence of light, the culture medium was removed, and 100 μL of MTT (0.5 mg/mL) was added to the cells. After 3.5 h (at 37 °C, 5% CO_2_, and 95% humidity), the MTT solution was removed, and the formed formazan crystals were dissolved in 100 μL of dimethyl sulfoxide (DMSO). The resulting purple color indicates the amount of formazan produced, which was measured at 570 nm using a spectrophotometer with a microplate reader (EZ Read 400, Microplate Reader, Biochrom, Holliston, MA, USA).

### 2.7. Contractility Experiments in HUASMCs

Cell contractility was assessed using the Planar Cell Surface Area (PCSA) technique following the method described previously by Cairrao’s research group [42]. This technique examines changes in cell surface area by capturing images of the cells and analyzing any decrease or increase in the cell area showing cell contractility or relaxation, respectively.

Briefly, HUASMCs were trypsinized and transferred into 6-well culture plates. Once the cells reached confluence, the complete culture medium was replaced with a serum-free medium consisting of DMEM-F12, 0.25% BSA, and the antibiotic mixture previously described. To conduct studies on the direct effects of DBP, the serum-free medium was placed for 48 h. Regarding the genomic studies, the cells were cultured in 6-well plates using glass coverslips (diameter of 30 mm) to avoid any potential reactions between the DBP and the polystyrene of the plates. HUASMCs were kept in a serum-free medium for 24 h before treatment with DBP at concentrations of 0.001, 0.1, and 100 μM, or to 0.01% solvent, for another 24 h. Both studies were conducted at 37 °C with 5% CO_2_ and 95% humidity.

After this incubation period, the cells were trypsinized and transferred into specific Petri dishes previously coated with collagen and incubated under the same conditions for 2 h. After that, the serum-free medium was removed, and the cells were washed four times with 500 μL of RCS modified solution, consisting of 124 mM NaCl, 5 mM HEPES, 10 mM TEA, 6 mM glucose, 5 mM CaCl_2_, and 4.7 mM KCl, and taken to the microscope.

The cells were observed through an inverted fluorescence microscope (Zeiss Axio Observer Z1, Jena, Germany). Microphotographs of groups of 2 or more cells were taken throughout each experiment with a high-speed digital monochrome Axio Cam Hsm camera (Zeiss, Jena, Germany). After the initial microphotograph, the contractile agent 5-HT (1 μM) was added and acted for 20 min, the necessary time to achieve a maximal response, during which the effect of the contractile agent reaches a plateau phase [42]. Additionally, during these 20 min, photographs are taken every 2 min to analyze the contractile profile of the cells. Then, to investigate the direct effects of DBP, different concentrations (0.001, 0.01, 0.1, 10, and 100 μM) were analyzed and microphotographs were taken both before and 20 min after the addition of each concentration. In the genomic studies, upon 24 h incubation with DBP (0.001, 0.1, and 100 μM), the cells were first treated with 5-HT (1 μM), following the vasorelaxant agents SNP at 100 μM or Nif at 10 μM. Photographs were taken over a 20 min period, and an additional photograph was captured at the end of this duration. Control experiments were also conducted using the percentage of ethanol (0.01%) corresponding to the maximum concentration of the DBP treatment, for the non-genomic as well as the genomic effects.

The analysis of cell area measurements was conducted using the “Automatic Measurement Program” supplement of the Axionvision 4.8 software (Zeiss, Jena, Germany). To ensure data variability, for each concentration this study was performed in triplicate for every human umbilical cord used (at least 3).

### 2.8. Drugs and Chemicals

Several chemical compounds were used in this research, specifically DBP, ethanol, 5-HT, Nif, and SNP, all purchased from Sigma-Aldrich Química (Sintra, Portugal). Stock solutions were prepared for each chemical, specifically, DBP and Nif were dissolved in pure ethanol, while 5-HT and SNP in Milli-Q water. All these chemical compounds were stored at −20 °C. During the several experiments performed, dilutions of these compounds were prepared everyday using different solutions: for the arterial contractility experiments, the Krebs’ solution was used; for the cell contractility tests, the dilutions were prepared in RCS and serum-free medium; and for the MTT assays, complete culture medium was used. To avoid interfering with the results, the final percentage of ethanol in all dilutions did not exceed 0.1%.

### 2.9. Statistical Analysis

The data obtained are presented as the mean ± standard error of the mean (SEM) of n independent experiments. Statistical analyses were conducted using the SigmaPlot Statistical Analysis System, version 15.0 (2022), while graphical representations were created using Origin version 9.8.0.200 (2021) software. To statistically analyze the results different tests were used. The Kolmogorov–Smirnov and Levene tests were used for the normality and homogeneity of variances, respectively. For the cell viability studies, the analysis was performed using the non-parametric Kruskal–Wallis One-Way ANOVA test. For the direct vascular effects of DBP on HUA contractility, the statistical analysis was also performed using the non-parametric Kruskal–Wallis One-Way ANOVA test and a Student’s *t*-test or the non-parametric Mann–Whitney test to analyze between solvent-treated and DBP-treated arteries. To analyze the long-term DBP exposure on HUA tension One-Way ANOVA followed by Dunn’s post hoc test was employed. Regarding the effects of DBP on the cGMP signaling pathway and the activity of LTCC, the analysis was conducted using a Two-Way ANOVA test followed by the Holm–Sidak post hoc test. For the cell contractility studies, a One-Way ANOVA test was performed followed by Holm–Sidak or Dunn’s post hoc tests regarding the direct effects of DBP and the long-term exposure, respectively. And lastly, the effects of DBP on the cGMP signaling pathway and the activity of LTCC of HUASMC was analyzed using Student’s *t*-test. Differences were considered statistically significant when the probability levels were less than 5% (*p* < 0.05). In the graphic representation, statistical significance is indicated as follows: p* < 0.05, p** < 0.01, p*** < 0.001; *p*# < 0.05, *p*## < 0.01, *p*### < 0.001.

## 3. Results

### 3.1. Cell Viability Studies

**Figure 1:**
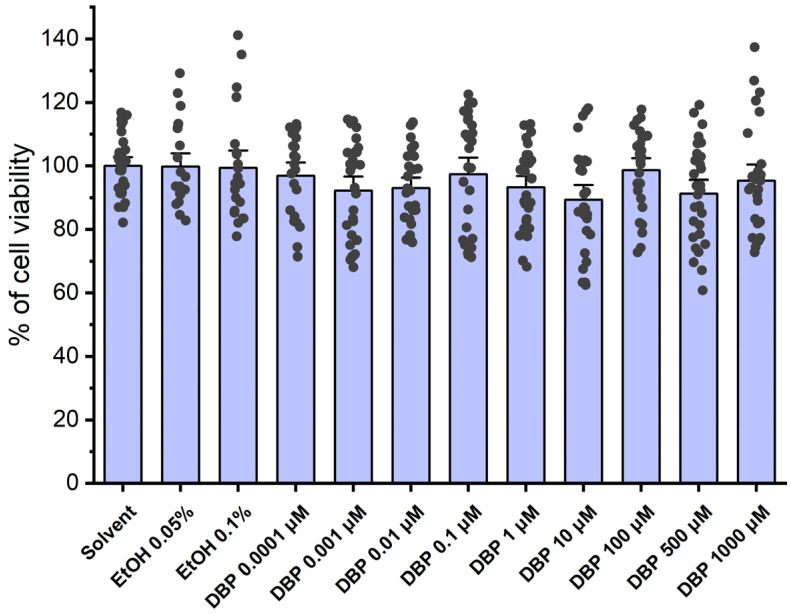
Percentage (%) of cell viability of HUASMCs after exposure to DBP. The bars represent the mean values, the vertical lines the SEM, and the individual dots the replicates of 9 independent experiments with different HUCs.

The viability of HUASMCs was assessed using the MTT assay after a 24 h incubation with several concentrations of DBP (0.0001; 0.001; 0.01; 0.1; 1; 10; 100; 500; and 1000 μM), as well as with solvent (ethanol 0.1% and 0.05%) and culture medium was used as negative control. Figure 1 summarizes the results obtained throughout the experiments. The analysis revealed that there were no statistically significant differences among any of the tested DBP concentrations, indicating that DBP did not induce overt cell toxicity.

### 3.2. Arterial Contractility Studies

#### 3.2.1. Direct Vascular Effects of DBP on HUA Contractility Induced by 5-HT and KCl

**Figure 2:**
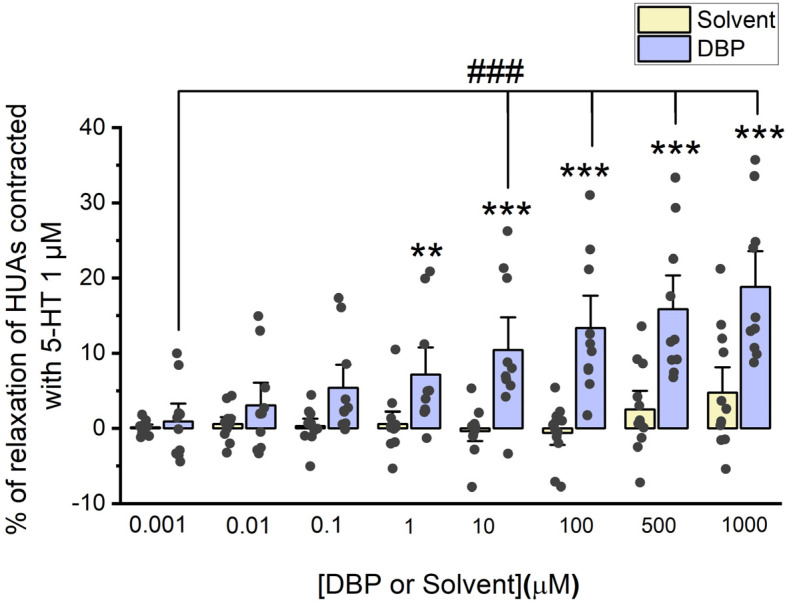
Evaluation of DBP effect on HUA contractions. The results are expressed as the percentage (%) of relaxation after contraction with 5-HT (1 μM), using 7 different HUCs for both the solvent- and DBP-treated groups. The bars represent the mean values, the vertical lines the SEM, the individual dots the replicates of each experiment, the symbol * the significant differences between both groups, and the symbol # the significant differences among DBP concentrations.

**Figure 3:**
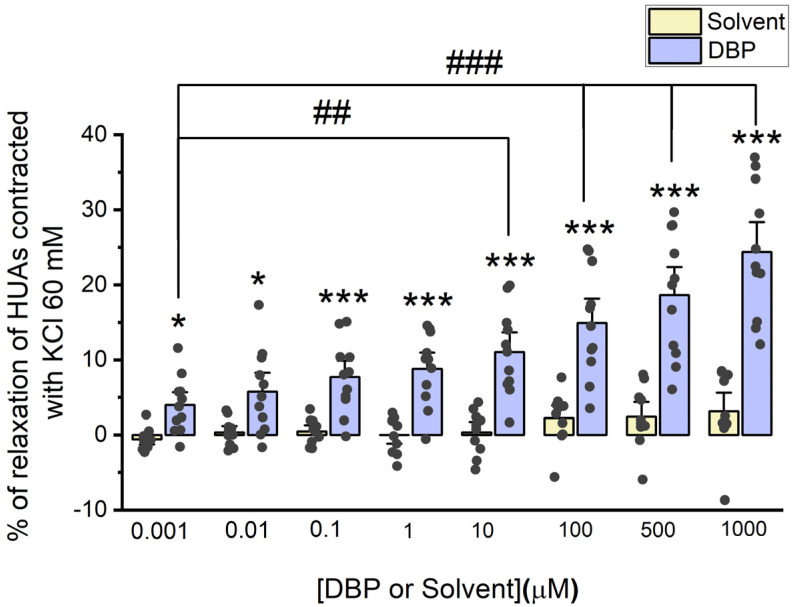
Evaluation of DBP effect on HUA contractions. The results were expressed as percentage (%) of relaxation after contraction with KCl (60 mM), using at least 6 different HUCs for both the solvent- and DBP-treated groups. The bars represent the mean values, the vertical lines the SEM, the individual dots the replicates of each experiment, the symbol * the significant differences between both groups, and the symbol # the significant differences among DBP concentrations.

The direct effects of DBP (0.001, 0.01, 0.1, 1, 10, 100, 500, and 1000 μM) were analyzed on the vascular rings of HUAs previously contracted using 5-HT (1 μM) and KCl (60 mM). The results from these experiments are summarized in Figure 2 and Figure 3.

The results of Figure 2 indicate that DBP induces vasorelaxation of the arterial rings, in a concentration-dependent manner. Differences were observed between the solvent-treated arteries and those treated with DBP at the following concentrations: 1 μM (*p* = 0.008), 10 μM (*p* < 0.001), 100 μM (*p* < 0.001), 500 μM (*p* < 0.001), and 1000 μM (*p* < 0.001). As anticipated, there were no significant differences among the solvent-treated group. In addition, differences were noted between the lowest (0.001 μM) and the subsequent DBP concentrations—10 μM (*p* < 0.001), 100 μM (*p* < 0.001), 500 μM (*p* < 0.001), and 1000 μM (*p* < 0.001).

DBP also induces vasorelaxation in arterial rings contracted with KCl, and this effect increases with DBP concentrations. The maximum effect is observed at the highest concentration of DBP (1000 μM), as illustrated in Figure 3. Differences were found between the solvent- and DBP-treated arteries across all tested concentrations: 0.001 μM (*p* = 0.042), 0.01 μM (*p* = 0.016), 0.1 μM (*p* = 0.001), 1 μM (*p* < 0.001), 10 μM (*p* < 0.001), 100 μM (*p* < 0.001), 500 μM (*p* < 0.001), and 1000 μM (*p* < 0.001). As expected, no significant differences were noted among the solvent-treated group. In addition, differences were observed between the lowest DBP concentration (0.001 μM) and the subsequent concentrations: 10 μM (*p* = 0.006), 100 μM (*p* < 0.001), 500 μM (*p* < 0.001), and 1000 μM (*p* < 0.001).

#### 3.2.2. Effects of Long-Term DBP Exposure on HUA Tension

**Figure 4:**
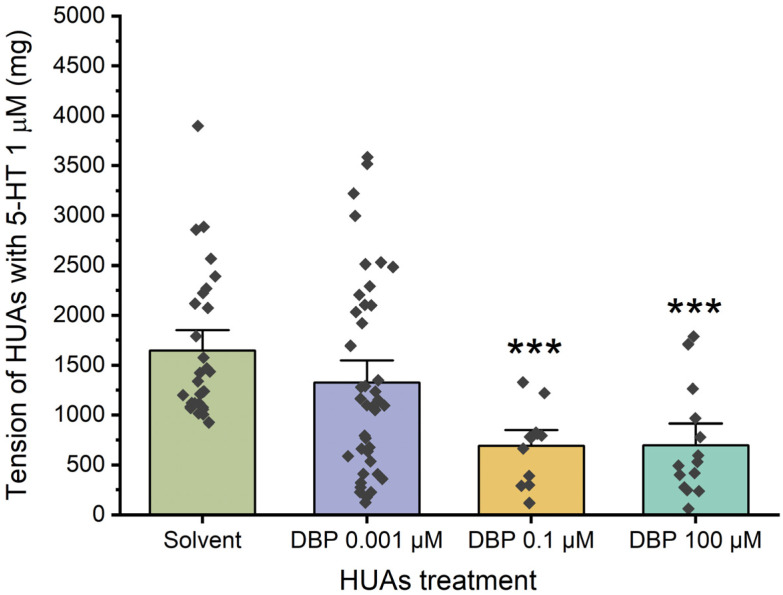
Tension (mg) upon 5-HT (1 μM) contraction of HUAs incubated with DBP (0.001, 0.1, 100 μM). At least 4 different HUCs were used for both solvent- and DBP-treated groups. The bars represent the mean values, the vertical lines the SEM, the individual dots the replicates of each experiment, and the symbol * the significant differences between both groups.

Regarding the genomic effects of DBP, the HUAs were incubated for 24 h with three different concentrations of the compound: 0.001 μM, 0.1 μM, and 100 μM. Additionally, a control group was treated with 0.01% ethanol, which corresponds to the maximum concentration used. Then, the arterial tension following contraction with 5-HT was analyzed, as illustrated in Figure 4. There is a significant decrease in the arteries incubated with DBP 0.1 μM (*p* < 0.001) and 100 μM (*p* < 0.001) compared to the solvent-treated arteries.

**Figure 5:**
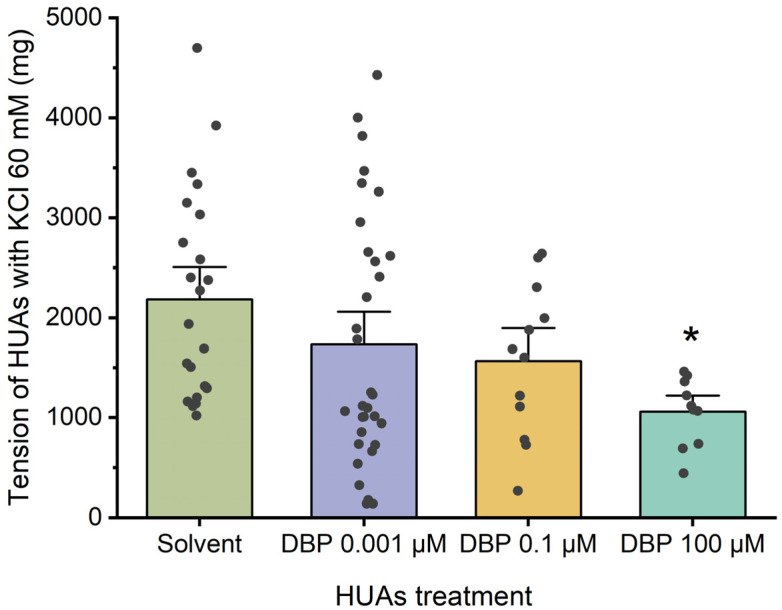
Tension (mg) upon KCl (60 mM) contraction of HUAs incubated with DBP (0.001, 0.1, 100 μM). At least 4 different HUCs were used for both solvent- and DBP-treated groups. The bars represent the mean values, the vertical lines the SEM, the individual dots the replicates of each experiment, and the symbol * the significant differences between both groups.

Figure 5 illustrates the arterial tension following contraction with KCl. In this case, a decrease was only observed at the highest concentration of DBP, 100 μM (*p* = 0.013), compared to the solvent-treated group.

The solvent-treated arteries were also compared with untreated arteries (not exposed to solvent nor DBP), either for 5-HT and KCl contraction, and no differences were observed (Figure S1 in Supplementary Material).

#### 3.2.3. Effects of DBP on the cGMP Signaling Pathway of HUAs

**Figure 6:**
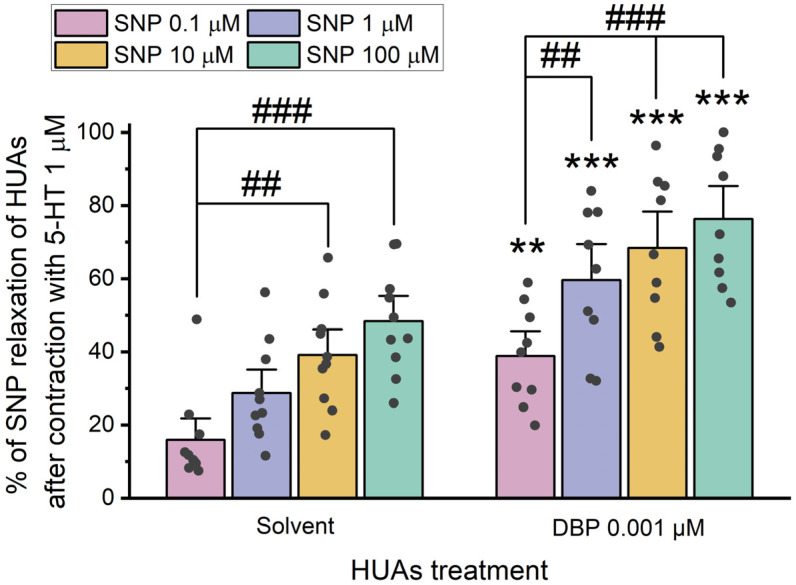
Percentage (%) of SNP relaxation of HUAs incubated with DBP (0.001 μM) after contraction with 5-HT (1 μM). At least 6 different HUCs were used for both solvent- and DBP-treated groups. The bars represent the mean values, the vertical lines the SEM, the individual dots the replicates of each experiment, the symbol * the significant differences between both groups, and the symbol # the significant differences among the SNP concentrations.

**Figure 7:**
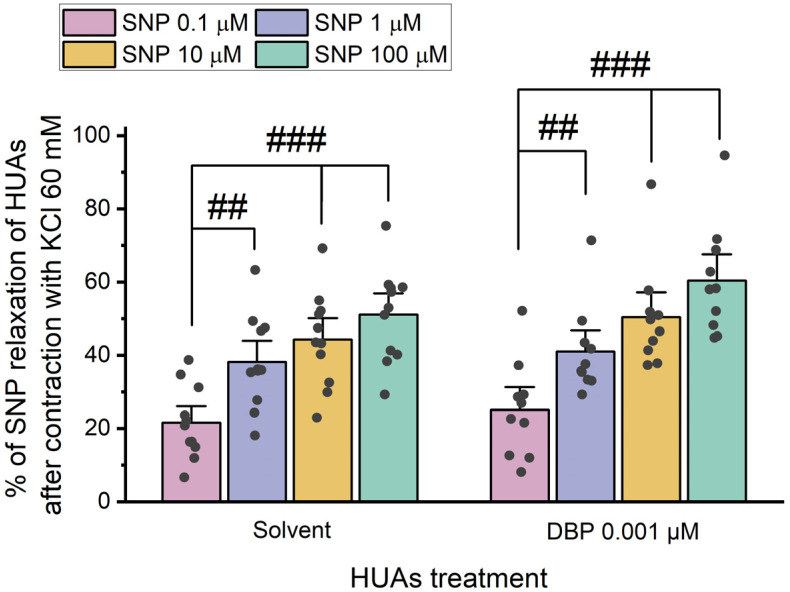
Percentage (%) of SNP relaxation of HUAs incubated with DBP (0.001 μM) after contraction with KCl (60 mM). At least 6 different HUCs were used for both solvent- and DBP-treated groups. The bars represent the mean values, the vertical lines the SEM, the individual dots the replicates of each experiment, and the symbol # the significant differences between the SNP concentrations.

After 24 h incubation with 0.001 μM DBP, the role of cGMP in the vasorelaxation was analyzed by adding different concentrations of SNP (0.1, 1, 10, and 100 μM) upon HUA contraction with 5-HT and KCl (Figure 6 and Figure 7).

The results presented in Figure 6 indicate differences between the solvent- and DBP-treated groups for all SNP concentrations: 0.1 μM (*p* = 0.002), 1 μM (*p* < 0.001), 10 μM (*p* < 0.001), and 100 μM (*p* < 0.001). Additionally, differences were observed between the lowest and the 10 μM (*p* = 0.003) and 100 μM (*p* < 0.001) SNP concentrations within the solvent-treated group. Likewise, in the DBP-treated group significant differences were noted between the lowest and the remaining concentrations of SNP: 1 μM (*p* = 0.007), 10 μM (*p* < 0.001), and 100 μM (*p* < 0.001). The solvent-treated arteries were also compared with untreated arteries (not exposed to solvent nor DBP), either for 5-HT and KCl contraction, and no differences were observed (Figure S2 in Supplementary Material).

The results presented in Figure 7 show no statistically significant differences between the solvent- and DBP-treated groups for all the SNP concentrations. However, within the solvent-treated group, there are significant differences between the lowest and the remaining SNP concentrations [1 μM (*p* = 0.004), 10 μM (*p* < 0.001), and 100 μM (*p* < 0.001)], as for the DBP-exposed group [1 μM (*p* = 0.007), 10 μM (*p* < 0.001), and 100 μM (*p* < 0.001)].

Overall, HUA rings treated with DBP demonstrated a more significant vasorelaxation in response to SNP upon contraction with 5-HT than KCl. The data on the role of cGMP indicate that a long-term HUAs exposure to DBP results in a significant increase in vasorelaxation after contraction with 5-HT.

#### 3.2.4. Effects of DBP on the Activity of L-Type Ca^2+^ Channels of HUAs

Similarly, the role of calcium channels in the vascular response triggered by DBP was examined using Nif, a specific inhibitor of L-type calcium channels (LTCCs).

**Figure 8:**
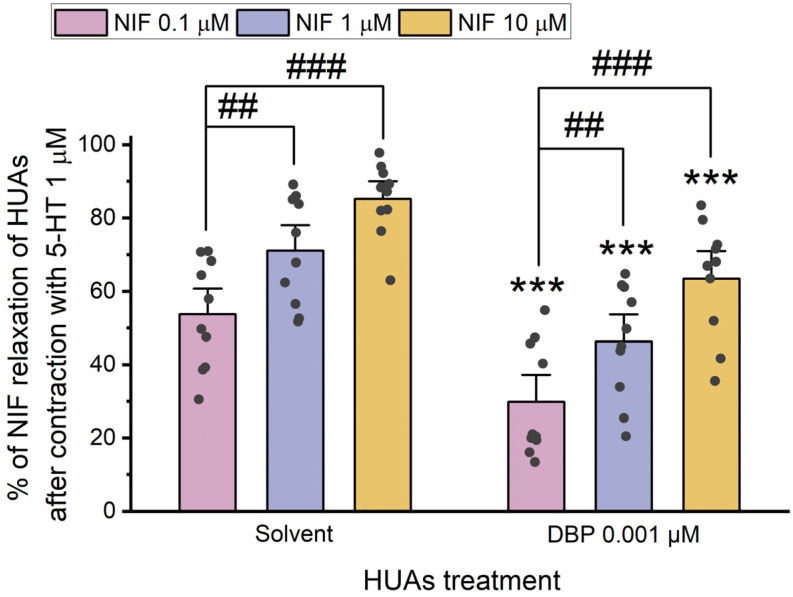
Percentage (%) of Nif relaxation of HUAs incubated with DBP (0.001 μM) after contraction with 5-HT (1 μM). At least 6 different HUCs were used for both solvent- and DBP-treated groups. The bars represent the mean values, the vertical lines the SEM, the individual dots the replicates of each experiment, the symbol * the significant differences between both groups, and the symbol # the significant differences among the Nif concentrations.

**Figure 9:**
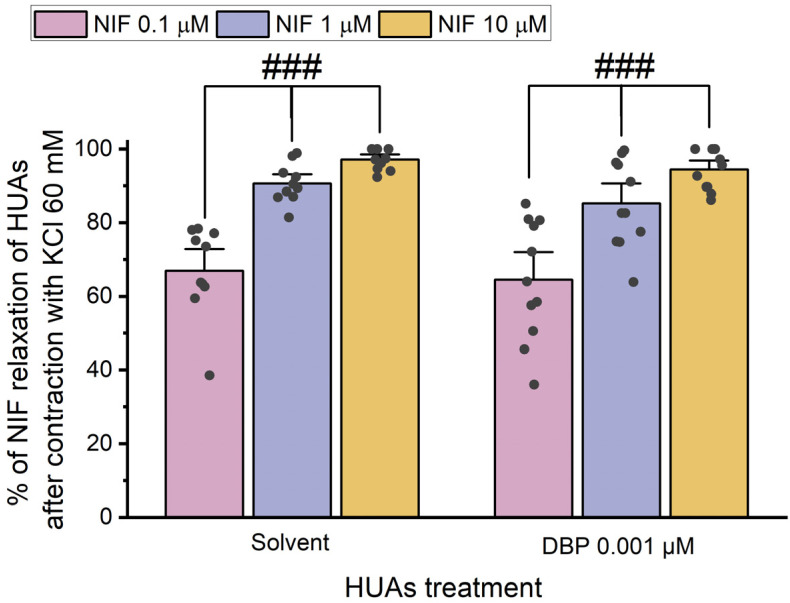
Percentage (%) of Nif relaxation of HUAs incubated with DBP (0.001 μM) after contraction with KCl (60 mM). At least 6 different HUCs were used for both solvent- and DBP-treated groups. The bars represent the mean values, the vertical lines the SEM, the individual dots the replicates of each experiment, and the symbol # the significant differences among the Nif concentrations.

After contraction with 5-HT and KCl, the HUA rings were exposed to different concentrations of Nif (0.1 μM, 1 μM, and 10 μM). The results of these experiments are summarized in Figure 8 and Figure 9.

The results indicate a decrease in vasorelaxation of the arteries incubated with DBP compared to the solvent-treated group. This difference was statistically significant for all concentrations of Nif tested: 0.1 μM (*p* < 0.001), 1 μM (*p* < 0.001), and 10 μM (*p* = 0.001). Additionally, Figure 8 shows differences between the lowest and the remaining Nif concentrations in the solvent-treated group [1 μM (*p* = 0.010) and 10 μM (*p* < 0.001)], as well as within the DBP-exposed group [0.1 μM of Nif with 1 μM (*p* = 0.014) and 10 μM (*p* < 0.001)]. The solvent-treated arteries were also compared with untreated arteries (not exposed to solvent nor DBP), either for 5-HT and KCl contraction, and no differences were observed (Figure S3 in Supplementary Material).

The results shown in Figure 9 indicate that there are no significant differences between the solvent- and DBP-treated groups at any of the Nif concentrations. However, differences were observed between the lowest and the other Nif concentrations in the solvent-treated group [1 μM (*p* < 0.001) and 10 μM (*p* < 0.001)], and in the DBP-treated group [1 μM (*p* < 0.001) and 10 μM (*p* < 0.001)].

Overall, when compared to the solvent-treated group, the HUA rings incubated with DBP exhibited a less pronounced vasorelaxation of Nif after contraction with 5-HT, while for the KCl-induced contraction, Nif promoted a similar vasorelaxation to the solvent-treated group.

### 3.3. Cell Contractility Studies

#### 3.3.1. Direct Vascular Effects of DBP on 5-HT-Induced Contractility of HUASMCs

**Figure 10:**
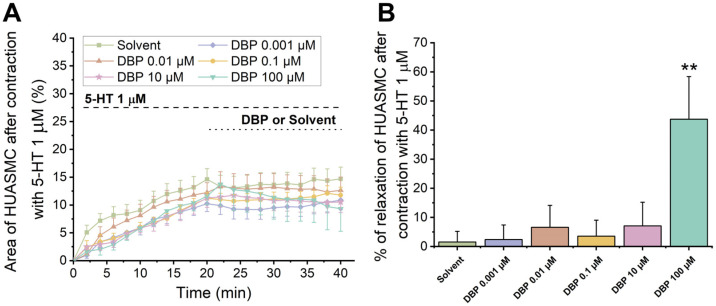
Direct effects of DBP (0.001, 0.01, 0.1, 10, and 100 μM) on HUASMC previously contracted with 5-HT (1 μM). (**A**) Time profile of 5-HT-induced contraction followed by relaxation induced by DBP in HUASMC. The data are presented as a percentage (%) of the compensated area reduction, the symbols indicate the mean values, and the vertical lines the SEM. (**B**) Percentage of relaxation of DBP on HUASMC previously contracted with 5-HT after a 20 min exposure. The bars represent the mean values, the vertical lines the SEM, and the symbol * significant differences between solvent- and DBP-treated groups. Data include at least 8 individual cells from 3 different HUCs.

Figure 10 summarizes the direct effects of DBP (0.001 μM, 0.01 μM, 0.1 μM, 10 μM, and 100 μM) at the cellular level. In Figure 10A, a temporal profile of the effects of 5-HT (1 μM) followed by DBP is shown. Figure 10B represents the effects of DBP on HUASMC after contraction with serotonin, where a significant difference was found between the solvent- and DBP-treated groups at the highest concentration, 100 μM (*p* = 0.002), indicating that DBP induced relaxation of HUASMC.

#### 3.3.2. Effects of Long-Term Exposure to DBP in the 5-HT-Induced Contractility of HUASMCs

**Figure 11:**
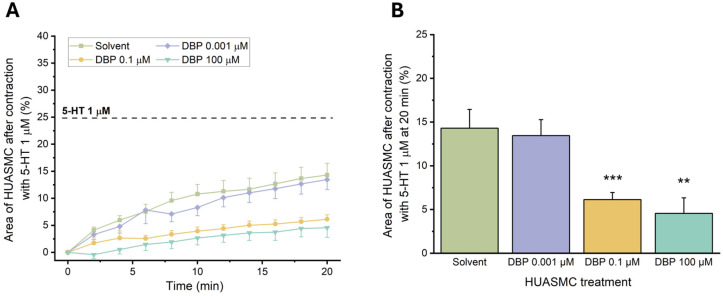
Effects of 24 h exposure to DBP (0.001, 0.1, or 100 μM) after HUASMCs 5-HT (1 μM) contraction. (**A**) Time profile of 5-HT-induced contraction in HUASMCs after incubation with DBP. The data are presented as a percentage (%) of the compensated area reduction, the symbols indicate the mean values, and the vertical lines the SEM. (**B**) Effects on the area of the HUASMCs after 5-HT contraction (20 min). The results are expressed as the percentage of the compensated area reduction. The bars represent the mean values, the vertical lines the SEM, and the symbol * significant differences between solvent- and DBP-treated groups.

To evaluate the genomic effects of DBP on the vascular reactivity, the HUASMCs were incubated for 24 h with the following concentrations: 0.001 μM, 0.1 μM, or 100 μM. Afterward, the cells were contracted using 5-HT (1 μM), and the results are shown in Figure 11.

There was a noticeable reduction in the compensated area for the cells exposed to 0.1 μM (*p* = 0.001) and 100 μM (*p* = 0.002) of DBP compared to the solvent-treated group. These results indicate that exposure to these concentrations significantly impaired the contraction induced by 5-HT in HUASMC. Therefore, subsequent analyses were conducted only with the lowest concentration of DBP.

#### 3.3.3. Effects of DBP on the cGMP Signaling Pathway and the Activity of LTCC of HUASMC

**Figure 12:**
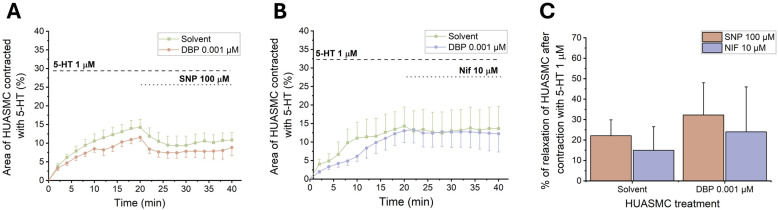
Effects of 24 h exposure to DBP (0.001 μM) on the HUASMCs. Time profile of HUASMCs contraction induced by 5-HT (1 μM) followed by relaxation induced by (**A**) SNP (100 μM) or (**B**) Nif (10 μM). The data are expressed as a percentage (%) of compensated area reduction, the symbols indicate the mean values, and the vertical lines the SEM. (**C**) Vasorelaxant effects of SNP and Nif in HUASMC contracted with 5-HT. The results are expressed as the percentage of relaxation. The bars represent the mean values, and the vertical lines the SEM. Data include at least 6 individual cells from 3 different HUCs.

To investigate the role of cGMP and Ca^2+^ channels in the cellular response induced by DBP, upon 24 h incubation of the HUASMC with 0.01 μM of DBP, the cells were subjected to SNP (100 μM) and Nif (10 μM). Figure 12A,B show the temporal profile of both SNP and Nif after contraction with 5-HT (1 μM), respectively, and Figure 12C the final vasorelaxant effect.

The results indicate a slight vasorelaxant increase after exposure to both 100 μM of SNP and 10 μM of Nif when compared to the solvent-treated group. However, none of these results achieved statistical significance.

## 4. Discussion

The use of plastic has dramatically increased over the past few decades, leading to greater human exposure to its toxic components, such as bisphenol A and phthalates. These compounds are known as endocrine disruptors because they interfere with hormonal balance [12]. DBP, a low molecular weight phthalate, is not covalently attached to plastic, which allows it to be easily released into the environment.

Human exposure to DBP can occur through various routes, mainly through ingestion and dermal exposure. It is important to note that infants and children may experience more severe health effects compared to adults due to their ongoing developmental processes [12]. In addition, long-last exposure to this type of compound can occur even before birth, as it has been shown that phthalates have the ability to cross the placental barrier, putting the infant at risk as early as the fetal period. Thus, pregnancy, as a period of hormone-mediated events, also becomes a window of increased susceptibility of exposure to EDCs, affecting the developing fetus as well as pregnant women [9].

Exposure to DBP has been implicated with adverse health effects, affecting mainly the reproductive system. Nevertheless, some studies have reported the effects of DBP on the cardiovascular system, indicating that this phthalate may promote the development of atherosclerosis in humans, increasing the risk of coronary disease [29,30,43]. In addition, research involving zebrafish has shown that exposure to DBP can lead to pericardial edema, structural and functional heart deformities, and a decrease in heart rate [44].

Taking these facts into consideration, the aim of this study was to investigate the potential effects of DBP on the cardiovascular system of pregnant women. Being widely used as a competent model to study the vascular effects of EDCs in pregnancy [9,45], we used umbilical cord arteries from pregnancies with no associated pathologies, and subsequent smooth muscle cells, to understand the genomic and non-genomic actions of DBP, either ex vivo and in vitro.

Cell viability was first assessed in HUASMC to evaluate the toxicity of DBP. When HUASMC were exposed to various concentrations of DBP (0.0001, 0.001, 0.01, 0.1, 1, 10, 100, 500, and 1000 μM), it was observed that none of these concentrations resulted in cell toxicity. This overt lack of toxicity may be attributed to the adsorption of DBP to the polystyrene material used in the 96-well plates for the MTT assays, since previous studies have indicated that the adsorption of DBP to polystyrene decreases the bioavailability of this compound [46,47,48], which is linked to a decrease in the toxicity of DBP [47]. These findings are consistent with the results obtained in the MTT assays conducted in this study. Thus, to prevent potential adsorption of DBP onto polystyrene, the use of polystyrene materials was minimized and replaced with glass whenever possible. Specifically, genomic studies were conducted using glass test tubes and by placing glass coverslips in the 6-well plates. In addition, the retention of another phthalate, DEHP, by the *Lycium barbarum* polysaccharides (LBPs) present in goji berries has already been demonstrated. Considering the contamination of drinking/tap water by this plasticizer and LBP, their interaction captures DEHP, reducing its free concentration and activity [49]. Although it has not yet been shown for DBP, to reduce the risk of contamination and false levels of free DBP, all the experiments were carried out using milliQ water.

When analyzing DBP actions on vascular contractility, the direct effects were studied both ex vivo and in vitro. For the ex vivo studies, control experiments were conducted using the solvent to dissolve DBP (ethanol), whose dilutions and final percentage did not exceed 0.1%. Despite the article by Rocha et al., in which the authors analyzed the effect of ethanol on the rat aorta, stating that it causes vasorelaxation through the NO/cGMP pathway [50], our data show that 0.1% ethanol has no effect on the vascular contractility of HUA, which was confirmed by comparing untreated arteries with arteries treated with the solvent. In addition, other studies that performed contractility experiments have also shown that this percentage of ethanol does not interfere with arterial or cellular contractility [51,52,53,54]. Then, after the contraction of the HUAs with 5-HT and KCl, it was observed that DBP induced vasorelaxation of the arterial rings, more pronounced at higher concentrations of the phthalate. Notably, vasorelaxation was more significant following contraction with KCl compared to that induced by 5-HT. Additionally, in vitro, DBP also led to vasorelaxation of the HUASMC following contraction with 5-HT. These data are in accordance with previous studies, also conducted on HUAs, that demonstrated a vasorelaxation by other EDCs [41,55,56]. Specifically, DEP, another low molecular weight phthalate, induced vasorelaxation following HUA’s contraction with KCl, but had no impact after 5-HT contraction [41], unlike DBP. On the other hand, other EDCs showed a more similar effect to DBP, since both BPA and OMC promoted vasorelaxation of the HUA, either after contraction with 5-HT or KCl [55,56].

Considering the ubiquitousness of phthalates, it is also important to explore the effects of long-term exposure to DBP. For this, both the arteries and the cells were exposed for 24 h to three different concentrations of DBP (0.001 μM, 0.1 μM, and 100 μM). Regarding the KCl-induced contraction only the incubation of 100 μM DBP resulted in a significant decrease in the vasoactive response of HUAs. On the other hand, following contraction induced by 5-HT in both the HUAs and the HUASMC, there was a notable reduction in contraction after long-term exposure to 0.1 μM and 100 μM of DBP. These results show that long-term exposure to intermediate and high levels of DBP have genomic consequences, since they led to a significant reduction in 5-HT contractions potentially by altering the serotonin receptors (5-HT_2A_ and 5-HT_1B_/5-HT_1D_). In contrast to the findings of this study, DEP did not show any changes in the tensions of the HUAs induced by 5-HT and KCl [41].

The primary mechanisms involved in the process of HUAs vasorelaxation include the NO/sGC/cGMP/PKG pathway, along with the activation or inhibition of ion channels [33]. SNP is an NO donor and an activator of soluble guanylate cyclase (sGC), promoting an increase in cGMP levels that will in turn activate protein kinase G (PKG), culminating in relaxation due to decreased intracellular Ca^2+^ concentrations [33,57]. Thus, the effect of SNP was studied on the contractility of HUAs and HUASMC previously incubated with 0.001 μM of DBP for 24 h. This specific concentration was chosen because it did not influence the contraction of either the cells or the artery. In the contraction induced by 5-HT, HUAs rings incubated with DBP exhibited a more pronounced vasorelaxation compared to the solvent-treated group, while those contracted with KCl showed similar vasorelaxation responses. The results from the in vitro assays demonstrated a slight increase in the relaxation of HUASMC incubated with DBP, although this was not statistically significant. Consistent with the findings of the present study, SNP had an enhanced vasorelaxant effect upon 24 h incubation with DEP following contraction with 5-HT; however, this effect was also observed after contraction with KCl [41]. The findings suggest that the mechanism of DBP may involve the cGMP/NO/sGC/PKG pathway. It is possible that the vasorelaxant effect of DBP on HUAs occurs through increased production of nitric oxide (NO). Several studies conducted on human endothelial cells have demonstrated that exposure to DBP results in enhanced NO production and increased migration of these cells [58,59]. This process appears to involve the G-protein-coupled estrogen receptor (GPER), the extracellular signal-regulated kinase 1 and 2 (ERK1/2), and NO signaling pathways [58,59]. Taking these results into account and considering that GPER is also highly expressed in VSMC, playing an important role in cardiovascular health [3,60,61], it is necessary to understand whether and how GPER is involved in DBP effects in VSMC. To determine GPER involvement, experiments should be conducted using known pharmacological tools, including GPER agonists (G1) and antagonists (G15 or G36) [61,62]. Considering that DBP has been suggested as an estrogenic compound [63,64], its involvement in GPER/ERK pathway is a distinct possibility since Masi et al. described that estrogenic substances can activate this signaling cascade via GPER [3]. However, other alternative pathways that may also be involved cannot be ruled out. For instance, receptor for activated C kinase 1 (RACK1), which is a scaffolding protein involved in several molecular pathways and cellular functions [4], has been proved as a relevant target of estrogenic active compounds [62]. In addition to estrogens, androgens have also been demonstrated to increase RACK1 expression, while glucocorticoids downregulate it, with regard to the immune system [62,65,66]. Therefore, considering that EDCs function similarly to hormones, it is possible that RACK1 may be a possible target for DBP mode of action. However, tissue-specific activity must be considered since the effects of DBP in HUASMC may be different.

Considering the involvement of the calcium channels in the contractile process, this study also evaluated the activity of LTCC as a key mechanism associated with the vasorelaxation of HUAs. To analyze the impact of DBP on this pathway, Nif was used as a specific inhibitor of LTCC, to further investigate how DBP affects the function of these channels. After exposure to the lowest concentration of DBP (0.001 μM), HUAs rings precontracted with 5-HT demonstrated a reduced Nif vasorelaxant effect, whereas those precontracted with KCl showed no differences compared to the solvent-treated group. Unlike the ex vivo findings, HUASMC contracted with 5-HT showed a slight increase in the vasorelaxant effect of Nif. However, this result was not statistically significant. The discrepancy in outcomes may be attributed to the fact that HUASMCs are more sensitive and less responsive compared to arteries, as in this case the cells exist isolated in a less physiologically relevant environment than the tissue.

Given that humans are constantly exposed to EDCs, including phthalates, it is important to analyze their effects after both acute and chronic exposure. A limitation of this work is the analysis of DBP effects after acute exposure of 24 h, only, although previous studies show that 24 h is enough time to induce genomic changes [34,67]. Therefore, in this study the rapid (non-genomic) action of different modulators (inductors/inhibitors of biological effects) after genomic effects was analyzed, i.e., how a 24 h exposure to DBP, which induces genomic changes, altered the rapid (non-genomic) action. However, this is also a limitation of this work, and molecular data could be useful in clarifying the genomic/non-genomic effects of DBP and the modulation of specific cell signaling pathways. In addition, as previously described, the range of DBP concentrations was also selected based on the non-monotonic dose–response curve. Although phthalates may present this type of curve, which is typical of EDCs [68,69], based on the results of this study, it is not possible to affirm that DBP has a non-monotonic profile regarding the vasoactive response of HUA. There were no inflection points or different effects at low or high concentrations that support this behavior (a U- or inverted U-shaped dose–response [68]), possibly due to the short duration of exposure to DBP. However, because it alters the normal contractile response, it is possible to suggest that DBP disrupts HUA vasoactivity.

These results align with the previously published study on DEP’s effects, showing that exposure to 0.01 μM of DEP decreased the Nif effect after HUAs were contracted with 5-HT [41]. However, opposite to the results observed with DBP, exposure to 0.01 μM and 100 μM of DEP after KCl-induced contraction of the HUAs led to a decrease in Nif vasorelaxation [41]. It appears that a 24 h exposure to DBP reduces the vasorelaxant capacity of Nif by interfering with LTCC. This disruption in calcium homeostasis caused by DBP may have significant implications for vascular health, potentially contributing to the development of cardiovascular diseases, namely pre-eclampsia. A possible connection between hypertensive disorders in pregnancy and phthalates exposure may be related to the modulation of RACK1 expression, particularly at the placental level. This protein, as mentioned previously, is involved in the inflammatory response, oxidative stress, cell migration, and angiogenesis, processes that are essential for normal placentation [70,71]. The dysregulation of these mechanisms can be induced by phthalates such as diethyl phthalate (DEP) [72], thus compromising the immune and vascular function of the placenta, promoting placental dysfunction and gestational hypertension. However, this possible link requires further study, where exposure to different phthalates, placentation, vascular contractility, gestational hypertension, and RACK1 should be analyzed.

Considering the constant human exposure to phthalates, several researchers have been testing the efficacy of different compounds as protective agents against phthalates. Most of these compounds present anti-inflammatory and antioxidant properties and have been mainly investigated for their protective actions in the reproductive system [73]. Polyphenols have been shown to counteract phthalates adverse effects, even in the cardiovascular system, since taxifolin alleviated DEHP effects in chicken cardiomyocytes [74,75,76]. In addition, as stated previously, *Lycium barbarum* polysaccharides (LBPs) has the ability to capture DEHP, reducing its free concentration and activity, and in rats has been shown to have potential detoxification effects against DEHP toxicity [49] and protected against oxidative stress induced by the combined exposure of DBP and DEHP of human hepatocellular carcinoma HepG2 cells [77]. Thus, as a future perspective, in addition to further exploring DBP mechanisms of action in vascular beds, including its effects on endothelial cells, either in acute and chronic exposure, it is intended to investigate the potential role of these compounds in protection or reversal of DBP adverse effects.

## 5. Conclusions

This study demonstrated that DBP does not induce overt cytotoxicity in HUASMC, potentially due to its adsorption onto polystyrene surfaces, which may reduce its bioavailability. Additionally, DBP exposure was shown to modulate contractile responses both at tissue and cellular level, affecting the HUAs and the HUASMC. The results suggest that, at the non-genomic level, DBP induces short-term, concentration-dependent relaxation, while at the genomic level, it altered the serotonin-mediated contractile capacity of both the HUAs rings and HUASMC. Furthermore, a 24 h exposure to DBP suggests an association with increased NO production and possible activation of the NO/sGC/cGMP/PKG signaling pathway, as well as an interference with LTCC, thereby attenuating the vasorelaxant response to Nif. Although the mechanisms have not yet been fully clarified, the results suggest the involvement of pathways associated with nitric oxide signaling and calcium handling. Should these results be confirmed, by disrupting the major pathways of vascular contraction and relaxation, exposure to DBP can potentially impair vascular homeostasis and contribute to the pathogenesis of cardiovascular diseases.

## Data Availability

The original contributions presented in this study are included in the article/supplementary material. Further inquiries can be directed to the corresponding author.

## Supplementary Materials

The following supporting information can be downloaded at: https://www.mdpi.com/article/10.3390/jox15040127/s1, Table S1: Total number of human umbilical cords (HUC) collected from the obstetrics unit of “Unidade Local de Saúde da Cova da Beira” (ULS Cova da Beira; Covilhã, Portugal) between 1 September 2023 and 31 October 2024. A total of 34 HUC were used in this study, 27 for the ex vivo analysis and 15 for cell culture (8 HUC were used for both situations).; Figure S1: Tension (mg) upon A) 5-HT (1 μM) and B) KCl (60mM) contraction of HUAs untreated and sol-vent-treated. At least 4 different HUCs were used for both the DBP and control groups. The bars represent the mean values and the vertical lines the SEM. Statistical analysis was performed using the Mann-Whitney Rank Sum Test.; Figure S2: Percentage (%) of SNP relaxation of HUAs untreated and solvent-treated after contraction with A) 5-HT (1 μM) and B) KCl (60 mM). At least 6 different HUCs were used for both groups. The bars represent the mean values, the vertical lines the SEM, the individual dots the replicates of each experiment. Statistical analysis was performed using a Two-Way ANOVA test followed by the Holm-Sidak post-hoc test.; Figure S3: Percentage (%) of NIF relaxation of HUAs untreated and solvent-treated after contraction with A) 5-HT (1 μM) and B) KCl (60 mM). At least 6 different HUCs were used for both groups. The bars represent the mean values, the vertical lines the SEM, the individual dots the replicates of each experiment. Statistical analysis was performed using a Two-Way ANOVA test followed by the Holm-Sidak post-hoc test.
