# Supplementary material for: The Plasticizer Dibutyl Phthalate (DBP) Impairs Pregnancy Vascular Health: Insights into Calcium Signaling and Nitric Oxide Involvement
Source: J Xenobiot. 2025 Aug 6;15(4):127. doi: 10.3390/jox15040127 (PMC12387933; doi:10.3390/jox15040127)
Supplement: Supplementary file 1 [file jox-15-00127-s001.zip › jox-3747653-supplementary.pdf]

Journal of
Xenobiotics

Supplementary Material

The Plasticizer Dibutyl Phthalate (DBP) Impairs Pregnancy Vascular Health: Insights into Calcium Signaling and Nitric Oxide Involvement

Ana R. Quelhas [1], Melissa Mariana [1,2,*] and Elisa Cairrao [1,2,*]

[1] RISE-Health, Department of Medical Sciences, Faculty of Health Sciences, University of Beira Interior, Av. Infante D. Henrique, 6200-506 Covilhã, Portugal; ana.rita.quelhas@ubi.pt
[2] FCS-UBI, Faculty of Health Sciences, University of Beira Interior, 6200-506 Covilhã, Portugal
* Correspondence: melissa.r.mariana@gmail.com (M.M.); ecairrao@fcsaude.ubi.pt (E.C.); Tel.: +351-275-329049

Table S1. Total number of human umbilical cords (HUC) collected from the obstetrics unit of "Unidade Local de Saúde da Cova da Beira" (ULS Cova da Beira; Covilhã, Portugal) between 1 September 2023 and 31 October 2024. A total of 34 HUC were used in this study, 27 for the ex vivo analysis and 15 for cell culture (8 HUC were used for both situations).

Pregnancy Criteria	Total	Number of HUC	
		Feminine	Masculine
No pathology	169	108	61
Gestational hypertension	3	2	1
Gestational diabetes	18	11	7
Hypo/Hyperthyroidism	3	2	1
Other	5	4	1
Unsupervised pregnancy	3	1	2
**Total used**	**34**	**22**	**12**

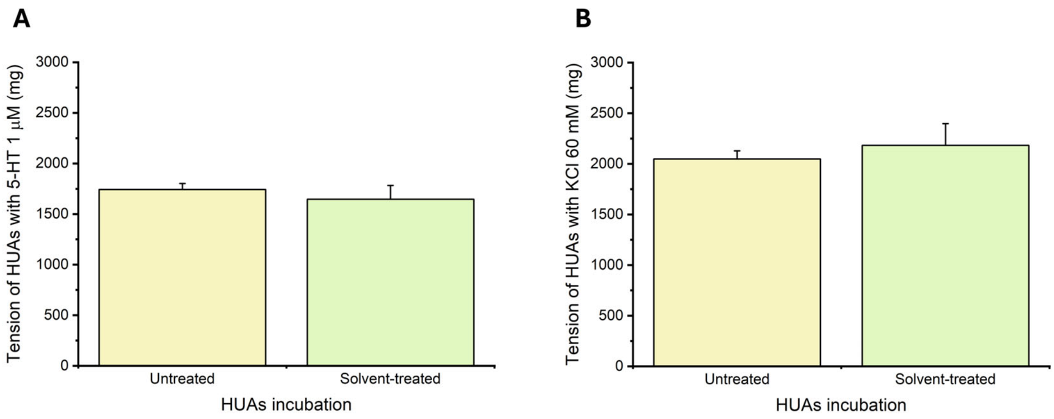

Figure S1. Tension (mg) upon **A)** 5-HT (1 μM) and **B)** KCl (60mM) contraction of HUAs untreated and solvent-treated. At least 4 different HUCs were used for both the DBP and control groups. The bars represent the mean values and the vertical lines the SEM. Statistical analysis was performed using the Mann-Whitney Rank Sum Test.

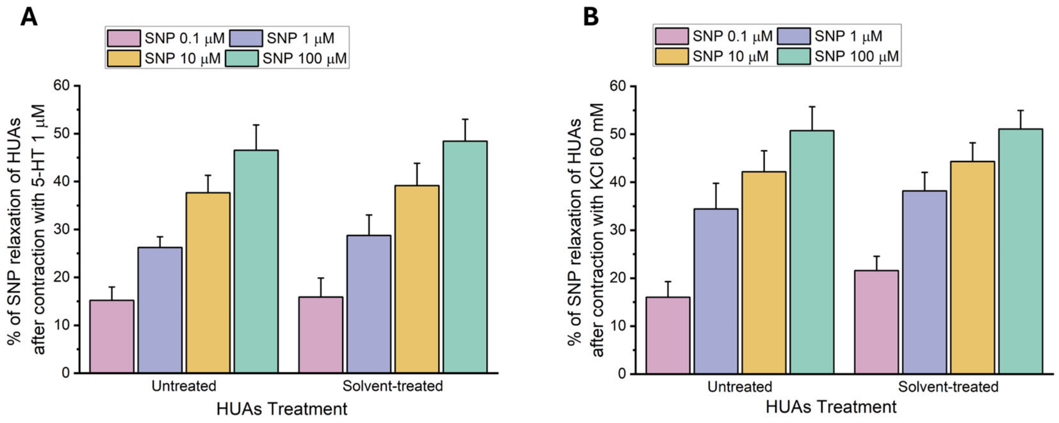

Figure S2. Percentage (%) of SNP relaxation of HUAs untreated and solvent-treated after contraction with A) 5-HT (1 μM) and B) KCl (60 mM). At least 6 different HUCs were used for both groups. The bars represent the mean values, the vertical lines the SEM, the individual dots the replicates of each experiment. Statistical analysis was performed using a Two-Way ANOVA test followed by the Holm-Sidak post-hoc test.

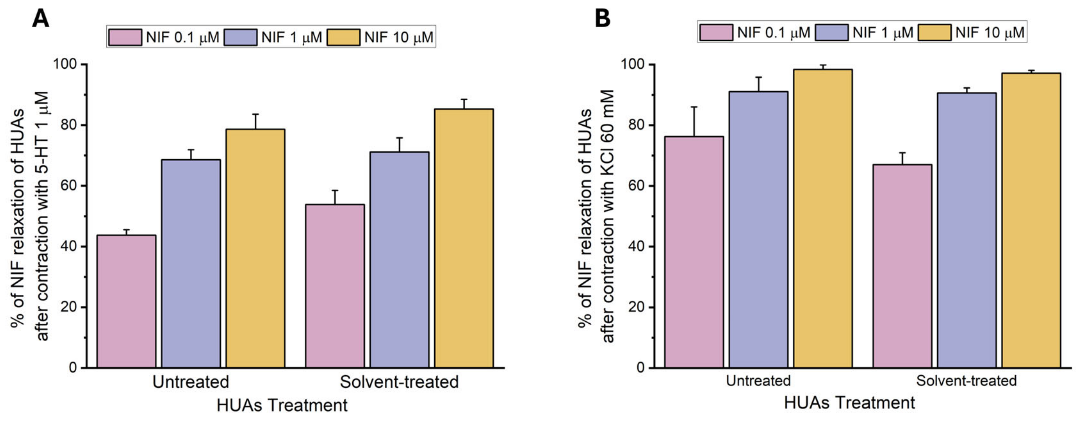

Figure S3. Percentage (%) of NIF relaxation of HUAs untreated and solvent-treated after contraction with **A)** 5-HT (1 μM) and **B)** KCl (60 mM). At least 6 different HUCs were used for both groups. The bars represent the mean values, the vertical lines the SEM, the individual dots the replicates of each experiment. Statistical analysis was performed using a Two-Way ANOVA test followed by the Holm-Sidak post-hoc test.
